# Effect of microserum environment stimulation on extraction and biological function of colorectal cancer stem cells

**DOI:** 10.1007/s12672-023-00779-z

**Published:** 2023-08-28

**Authors:** Feiqing Wang, Jianing Zhao, Chike Zhang, Bo Yang, Tingting Tian, Mengxian Tian, Na Meng, Wei Xie, Guangyang Liu, Xiaodong Zhu, Min Su, Zhixu He, Yang Liu, Dongxin Tang, Yanju Li

**Affiliations:** 1 Clinical Medical Research Center, The First Affiliated Hospital of Guizhou University of Traditional Chinese Medicine, No. 71 Bao Shan North Road, Yunyan District, Guiyang, 550001 Guizhou Province China; 2 Academy of Medical Engineering and Translational Medicine, Tianjin University, Tianjin City, 300072 China; 3 Department of Hematology Oncology, Affiliated Hospital of Guizhou Medical University, No. 4 Bei Jing Road, Yunyan District, Guiyang, 550004 Guizhou Province China; 4 Key Laboratory of Adult Stem Cell Translational Research, Chinese Academy of Medical Sciences, Guizhou Medical University, Guiyang, 550004 Guizhou Province China

**Keywords:** Microserum environment, Colon cancer tumor cells, Cancer stem cells, Tumor sphere enrichment

## Abstract

**Background:**

3D cancer stem cell (CSC) cultures are widely used as in vitro tumor models. In this study, we determined the effects of enriching HCT116 tumor spheres initially cultured in serum-free medium with different concentrations of serum, focusing on the effect of microserum environment stimulation on extraction and biological function of colorectal cancer stem cells (CCSCs).

**Methods:**

CCSCs were enriched in standard serum-free medium and serum-free medium with different concentrations of serum for 1 week. The expression of CSC-associated markers in CCSCs, and the presence and relative proportion of CSCs (CD133/CD44 cell sorting) were then determined to elucidate the effect of the microserum environment on the preservation of CSC-related features. Further, the tumorigenic capacity of CCSCs was evaluated in an immunodeficiency mouse model.

**Results:**

Our data indicated that a significantly greater number of spheres with a greater size range and high viability without drastic alteration in biological and structural features, which maintained self‐renewal potential after sequential passages were formed after serum supplementation. Real-time analysis showed that both serum spheres and serum-free spheres displayed similar expression patterns for key stemness genes. Serum spheres showed higher expression of the CSC surface markers CD133 and CD44 than did CSCs spheres cultured in serum-free medium. Adherent cultures in complete medium could adapt to the serum-containing microenvironment faster and showed higher proliferation ability. The addition of serum induced EMT and promoted the migration and invasion of serum globular cells. Compared with serum-free cells and adherent cells, serum spheres showed higher tumor initiation ability.

**Conclusions:**

Microserum environment stimulation could be an effective strategy for reliable enrichment of intact CCSCs, and a more efficient CSC enrichment method.

## Background

In recent years, cancer stem cells (CSCs) have been recognized as one of the major causes of cancer treatment failure and recurrence [1–3]. Many studies have shown that colorectal cancer stem cells (CCSCs) are a population of cells with stem cell-like characteristics in colorectal cancer tissues with the ability to self-renew, differentiate, proliferate, and replicate and are likely the main cause of tumor growth, metastasis, and recurrence, as well as the failure of chemotherapy and radiotherapy [4, 5]. Most colorectal cancer tumor cells can be killed during the treatment process. However, new tumors are formed by the CCSCs which are not removed, which eventually leads to treatment failure [6, 7].

A key step in the study of CSCs is the method of isolation. Three experimental methods are currently used to identify and sort CSCs: identification and isolation of dry cells expressing specific surface marker proteins (CD44, CD133, Lgr5, and EpCAM) using fluorescence-activated cell sorting (FACS) or magnetic-activated cell sorting (MACS) [8], analysis and isolation of side population (SP) cells by flow cytometry [9], and sphere-formation assays [10].

Sphere formation assay or non-adhesive 3D culture is the culture of tumor cell “spheres” in serum-free medium containing various growth factors [11]. It is a simple, low-cost method, and can enrich all phenotypes of CSCs to generate a cellular model for CSC research. The spheres can also be used in flow cytometry or magnetic bead sorting, and can circumvent technical problems in the sorting process to some extent [12]. Sphere formation is therefore the most widely used method to isolate and enrich CSCs and determine stemness potential [13]. In this method, suspended single cells are cultured at low cell density under nonadherent or ultra-low adherent suspension culture conditions or 3D culture, in serum-free (SF) conditions [13]. The addition of EGF, bFGF, and other cell growth factors can induce the growth of CSC suspension cultures into spheroids [14, 15]. Compared with regular adherent cultures, spheroids preserve in vivo tumor cell characteristics (including gene expression profiles, cellular heterogeneity, signal pathway activity, growth kinetics, tumor morphology, hypoxia and exposure to oxygen, nutrients, and metabolites) better, and include cells in different stages of development (proliferating, quiescent, apoptotic, hypoxic, and necrotic) that closely resemble in vivo conditions [16, 17].

Emerging evidence has revealed that the addition of specific serum under specific conditions during the extraction of MSCs or the enrichment of tumor stem cells can have a positive effect [18–20]. It has also been shown that it is entirely possible to cultivate CSCs from serum-containing media [21, 22]. In this study, we cultured HCT116 in ultra-low adhesion plates with serum-free medium containing EGF, FGF-2, and B-27. We then added different concentrations of FBS to observe its effect on sphere formation, cell proliferation, migration, and invasion, expression of CSC markers, and tumorigenicity.

## Methods

### Cell culture conditions

Cell culture of fluorescein-expressing cell lines HCT116-luc1 (CTCC) and HCT116 (ATCC) was performed using high-sugar Dulbecco’s modified Eagle medium (H-Glu DMEM). The medium was supplemented with fetal bovine serum (FBS, 10%) and 1% penicillin–streptomycin (Sorabio, Beijing, China) procured from Gibco (Rockville, MD, USA).

### Generation of HCT116 colon cancer spheroids

Cells (at a density of 5 × 10^3^ per 1 mL of medium) were seeded into 6-well ultra-low attachment plates (Corning Inc. Kennebunk, ME, USA) with serum-free DMEM supplemented with 20 ng/ml human epidermal growth factor (Gibco, MA, USA), 20 ng/mL human basic fibroblast growth factor (Abcam), 1 × B-27 supplement (Gibco, MA, USA), and 1% penicillin–streptomycin or serum-free medium containing 1%, 3%, 5% FBS for 7 days to form spheres.

### Sphere‐forming efficiency (SFE) assay

The culture medium from each well was collected in a 15 ml centrifuge tube on day 3, day 5, and day 7 and flicked. 50 μl of the cell suspension was then pipetted into 5 wells of a 96-well plate; this was repeated three times. Measure the size of the spheres in each well under a microscope (diameter of at least 50 μm). Then count the number of spheres in each well under the microscope (at least 50 μm in diameter). The average number of spheres was calculated and adjusted to the same volume to determine the total number of spheres. The total number of spheres can be calculated based on this simple formula: total number of spheres = (number of spheres in each well ÷ 50 μl/1 ml).

### Cell viability assay

HCT116 cells were seeded at a density of 2 × 10^3^ cells per well in 96-well plates (Corning Life Sciences, Union City, California, USA) with an ultra-low attachment surface, and the cells were cultured for 7 days using appropriate media, and the cell absorbance values were measured at fixation times after 3, 4, 5, 6, and 7 days using the CCK8 kit (Dojindo, Kumamoto, Japan), and growth curves were plotted.

### Flow cytometry

To assess the stem cell phenotype of hct116 spheroids, single-cell suspensions of serum spheres and serum-free spheres were incubated with the antibody mix CD133-APC and CD44-FITC or isotype control antibodies for 10 min at 2–8 ℃. And the cells were washed twice with pre-cooled PBS buffer and analyzed on a BD C6 Plus flow cytometer.

### Wound-healing assay

Single-cell suspensions of serum spheres and serum-free spheres were counted and inoculated into 6-well plates (10^6^/well) and incubated overnight in a cell incubator. Draw a straight line from the cell monolayer using a sterile suction head, cells were washed 2–3 times with PBS, fresh medium was added and incubated in the cell culture incubator and photographs of the scratch wounds of different samples were taken at 0, 24 and 48 h.

### Invasion assay

Primary cultures of colon spheres were collected after 7 days of culture and dissociated enzymatically and mechanically by using trypsin–EDTA and pipetting, respectively. The cells were washed with PBS and resuspended in serum‐free media. 3 × 10^4^ cells were seeded on Matrigel-coated inserts in 200 µl of serum-free medium inserts for invasion assays. The lower chambers were filled with 0.5 ml 10% FBS-supplemented DMEM medium. After 24 h, cells on the upper side of the filter were removed and the cells on the lower surface of the insert were fixed and stained with crystal violet. The number of stained cells was counted under a light microscope. Assays were performed in triplicates.

### Colony formation assay

Primary cultures of colon spheres were collected after 7 days of culture and dissociated enzymatically and mechanically using trypsin–EDTA and pipetting, respectively. Cells were disassociated, suspended, and plated in a 6-well culture plate at a density of 800 cells/well. After culturing for 14 days at 37 °C, the cells were washed twice with PBS and stained with crystal violet. Colonies larger than 75 μm in diameter or containing more than 50 cells were counted as positive colonies.

### EdU staining for cell proliferation

Cells were incubated with DMEM supplemented with 10 μm EdU (Cat.No.C10310, RiboBio, China) [23] for 24 h. Cells were then washed with phosphate-buffered saline (PBS), fixed in 4% paraformaldehyde, incubated with glycine 2 mg/ml, washed with PBS twice, and permeabilized with PBS containing 0.5% Triton X-100. After extensive washing with PBS, cells were incubated with Apollo^®^ staining solution for 30 min, washed with PBS containing 0.5% Triton X-100 three times, and incubated for 10 min with DAPI dye. Cells were then imaged under an inverted fluorescence microscope (Nikon, Tokyo, Japan). Three random positions in each well were photographed, and the fluorescence of EdU-positive cells was measured using ImageJ 1.8.0 software (NIH, Bethesda, MD, USA). Spheres are stained in the same way as the adherent cells, but the cells must be centrifuged (1000 rpm, 5 min, room temperature) and the supernatant removed before adding different reagents.

$$ {\text{EdU-positive cells }}\left( \% \right) \, = {\text{ red EdU count/blue Hoechst count}} \times 100. $$

### QPCR

For qPCR analysis, total RNA was extracted from the serum spheres and serum-free spheres by using TRIzol reagent (Solarbio, Beijing, China) according to the manufacturer’s instructions. Reverse transcription was performed using an EasyScript One-Step gDNA Removal and cDNA Synthesis SuperMix kit (TransGen Biotech, Beijing, China). QPCR was performed using a Top Green qPCR SuperMix kit (TransGen Biotech, Beijing, China) in a CFX96 Real‐Time PCR Detection System (Bio‐Rad). The sequences of the primers used are listed in Table 1.

**Table 1:** Nucleotide sequences of primers used for quantitative polymerase chain reaction analysis

Gene name	Forward (5′–3′)	Reverse (5′–3′)
GAPDH	GAAAGCCTGCCGGTGACTAA	GCCCAATACGACCAAATCAGAG
OCT-4	CTCGAGAAGGATGTGGTCCG	TAGTCGCTGCTTGATCGCTT
SOX2	AGGATAAGTACACGCTGCCC	TAACTGTCCATGCGCTGGTT
NANOG	AATGGTGTGACGCAGGGATG	TGCACCAGGTCTGAGTGTTC
ALDH	TGCCGGGAAAAGCAATCTGA	CAACAGCATTGTCCAAGTCGG
CD133	CGGGTGCACGGGATGG	TTCTGTCTGAGGCTGGCTTG
LGR5	AAGCCTTCAATCCCTGCGTC	CAGGCCACTGAAACAGCTTG
CD44	ACACAAATGGCTGGTACGTCT	TGTGGTTGAAATGGTGCTGG
EpCAM	GCTGGCCGTAAACTGCTTTG	ACATTTGGCAGCCAGCTTTG
E-cadherin	TACCCTGGTGGTTCAAGCTG	CAAAATCCAAGCCCGTGGTG
N-cadherin	ATGGGAAATGGAAACTTGATGGC	CAGTTGCTAAACTTCACTGAAAGG

### Western blot analysis

Protein lysates were extracted from the cultures by using RIPA buffer (Beyotime, Beijing, China) supplemented with protease inhibitors (Boster, China). The proteins (30 μg) were separated by 10% SDS-PAGE and transferred onto nitrocellulose membranes. The membranes were blocked with 5% non-fat milk for 2 h at room temperature, and incubated with specific primary antibodies at 4 °C overnight. The membranes were washed with ice-cold TRIS-buffered saline Tween (TBST) five times, then incubated with corresponding secondary antibodies for 2 h at room temperature. The specific antibodies used were: CD133 (ABclonal, NO. A0818, 1:1000), CD44 (ABclonal, NO. A12410, 1:1000), NANOG (Proteintech, No. 14295-1-AP, 1:1000), OCT4 (Proteintech, No. 60242-1-Ig, 1:1000), SOX2 (Proteintech, 66411-1-Ig, 1:1000), E-cadherin (Cell Signaling, No. 3195, 1:1000), N-cadherin (Cell Signaling, No. 13116, 1:1000), Vimentin (Cell Signaling, No. 5741, 1:1000), No. AF0141, 1:1000), CCL2/MCP-1 (Beyotime, No. AF7437, 1:1000), and IL-6 (Solarbio, No. K009341P, 1:1000), GAPDH (Beyotime, No. AF0006, 1:1000). The secondary antibodies were goat anti-rabbit IgG-HRP (Boster, No. BA1054, 1:5000) and goat anti-mouse IgG-HRP (Boster, No. BA1050, 1:5000). Protein bands were exposed and images were acquired using the ECL kit and the Burroughs imaging system, and the density of each strip was quantified using Image J software (ImageJ 1.48v, USA).

### In vivo tumorigenicity and histological analysis of tumors

Male BALB/c nude mice (4 weeks old, 20 ± 2 g) were housed in an SPF-grade animal laboratory for 7 days. To compare the tumorigenicity of serum-free spheres and serum spheres to that of HCT116 parental cells, mice were divided into five groups randomly (3 mice per group). All animal experiments were performed in the pathogen-free medical animal laboratory of the First Affiliated Hospital of Guizhou University of Traditional Chinese and were approved by the Animal Ethics Committee of the First Affiliated Hospital of Guizhou University of Traditional Chinese (approval number: AHQU20190455A). HCT116 cells were cultured in the same density and under the same conditions as the test cells (adherent culture in complete medium, suspension culture in serum-free medium, suspension culture in serum-free medium with different concentrations of fetal bovine serum). When the number of HCT116 cells cultured in adherent culture reached 1 × 10^6^, the cultures in all groups was terminated, and the cells in each group were counted after digestion. Single-cell suspensions were prepared from HCT116 parental cells, HCT116-luc1 serum-free spheres, and HCT116-luc1 serum spheres, mixed with PBS, and subcutaneously injected into the right armpit of nude mice. On the 7th day of cell inoculation, nude mice without tumorigenesis or with too large tumor tissue were removed. The activity and mental state of the nude mice, food and water consumption, and casualties were observed every day.

After taking the molded mice under isoflurane anesthesia, Luc1 substrate (DTZ) was injected intraperitoneally at a dose of 5 µl/g mouse. Luminescence imaging detection was performed by IVIS^®^Spectrum imaging system. And the tumor cell photon count was calculated using Living Image 3.1 software (Caliper) software analysis.

The BLAB/c nude mouse in colorectal cancer model was monitored using bioluminescence imaging (IVIS^®^Spectrum imaging system). The instrument used for photon detection can be found here: https://www.perkinelmer.com.cn/product/ivis-instrument-spectrum-120v-andor-c-124262.

### Statistical analysis

In the study, data are described as mean ± standard deviation (SD). Differences between two groups were assessed with the two-tailed Student’s t-test. Differences between multiple groups were evaluated with One-way ANOVA (LSD post hoc test). Statistical differences were indicated when *P* < 0.05 and significant differences were indicated when* P* < 0.01. GraphPad Prism 8.0 was used to analyze the data and create graphs.

## Results

### Microserum environment maintains the morphology of HCT116 spheroids

**Fig. 1:**
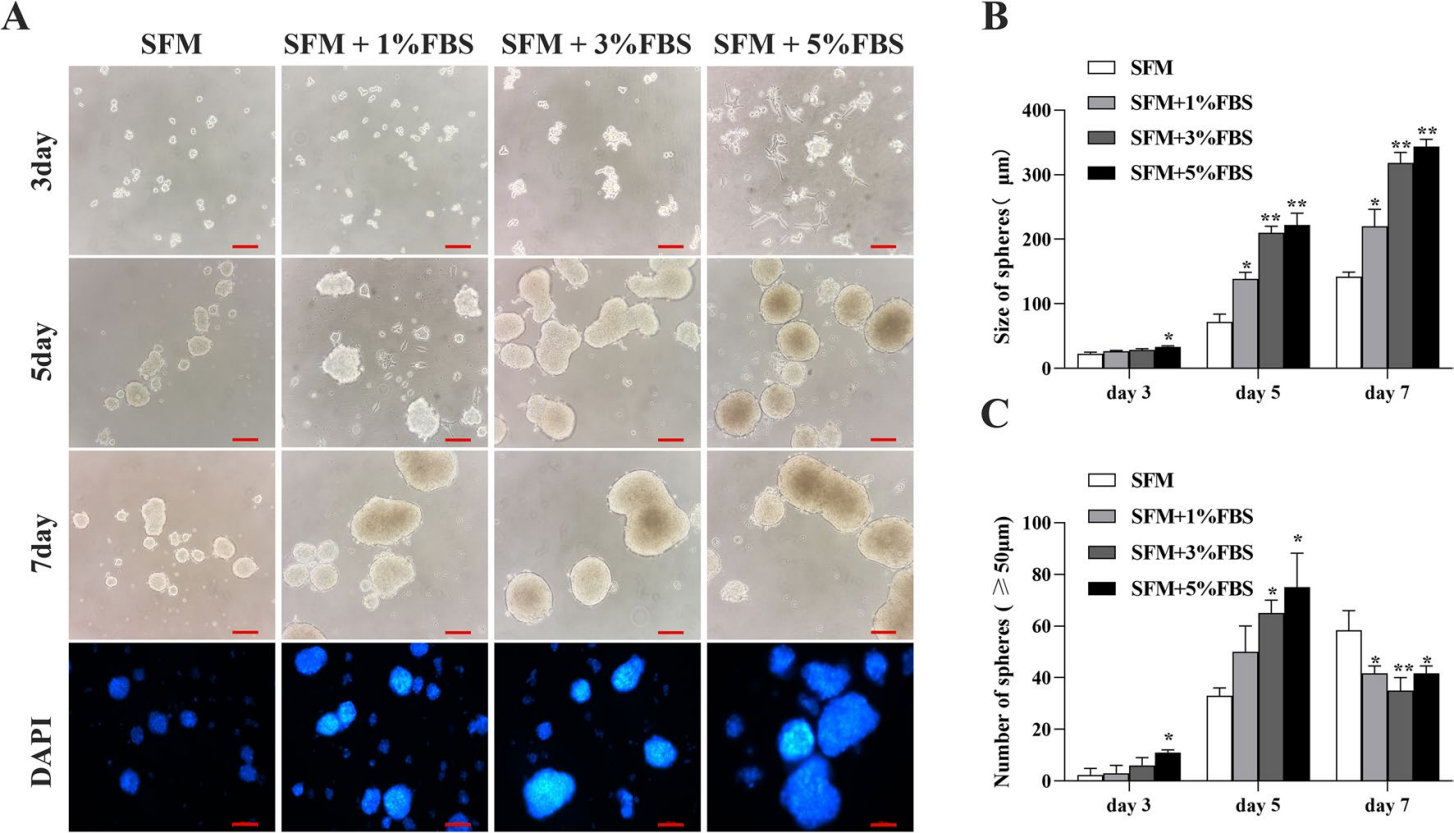
Primary spheroids obtained by enriching subpopulations of CCSCs in microserum environment: **A** Representative images and fluorescent staining of spheres derived from single-cell suspensions of HCT116 cells (5 × 10^3^ cells suspended in 1–2 ml serum-free medium) with 1–5% FBS. Bars: 100 μm. The number (**B**) and size (**C**) of primary established tumor spheres of the HCT116 cell line obtained by culture in serum-free medium and microserum environment. *P < 0.05; **P < 0.01 vs SFM

We established a culture method of adding microsera to conventional serum-free cultures for the suspension culture of CSCs (Fig. 1). At the initial stage of sphere enrichment, compared to the serum-free culture groups, there was no apparent variation in the number or size of spheroids formed in HCT116 colon cancer cell lines in serum-free medium supplemented with 0–3% FBS. In the culture system containing 5% FBS, some cells adhered to the six-well plate wall, and a small number of suspended spheres were produced (Fig. 1A). From the 5th day of culture, the sphere-forming rate of the tumor cells started to trend higher in the culture systems with added serum. On the 7th day, the larger spheroids first formed in the culture medium with 1–5% FBS, and showed a fusion trend. The number of independent cell spheroids suddenly decreased. However, larger spheroids with clearer outlines and closer to round shape were observed.

**Fig. 2:**
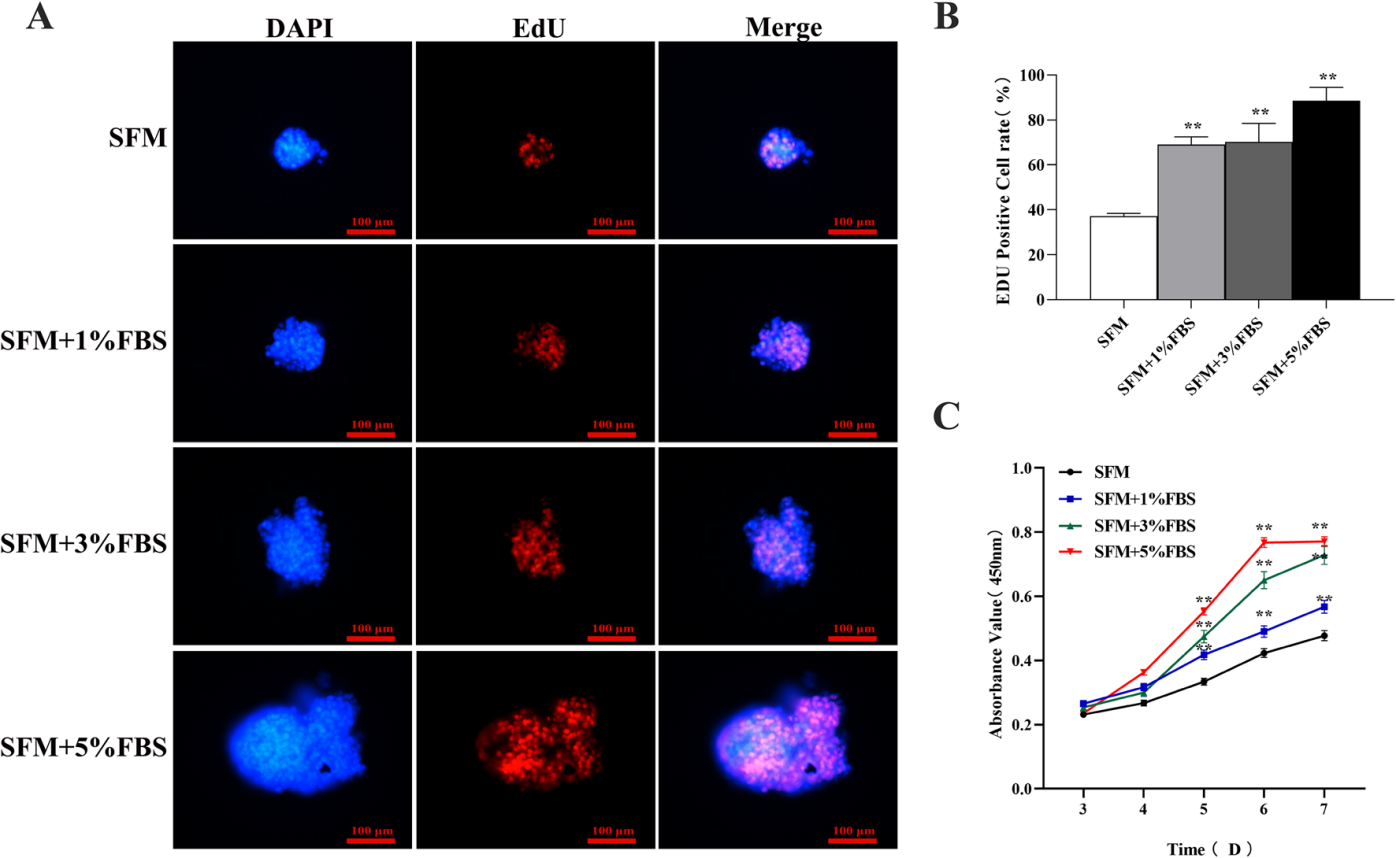
Comparison of growth of tumor spheres formed in the microserum environment: **A**, **B** The proliferation capacity of HCT116 cell spheres enriched for 7 days in microserum environment (0, 1%, 3%, and 5%) was measured using an EdU assay (× 200). **C** Cell Counting Kit-8 (CCK-8) analysis of human colorectal cancer (CRC) cell line HCT116 cell spheres after enrichment in microserum environment for 3, 4, 5, 6, and 7 days. *P < 0.05; **P < 0.01 vs SFM

To assess the effect of microserum environment on the core layer and overall structure of spheroids, DAPI staining was performed. Stimulation by micro serum did not lead to any disintegration of the globule structure, and the spheroids retained their intact spherical morphology and well-organized structure (Fig. 1A). The EdU assay showed that there was a higher percentage of EdU-positive cells in the serum culture spheroids (Fig. 2A). The histogram shows the corresponding proliferation rates (Fig. 2B). CCK-8 showed the highest absorbance values in the incubation system containing 5% FBS (Fig. 2C). Thus, HCT116 cells exhibited significantly higher sphere formation capacity when stimulated by the microserum environment.

### Microserum environment preserves the HCT116 spheroids CSC markers expression

**Fig. 3:**
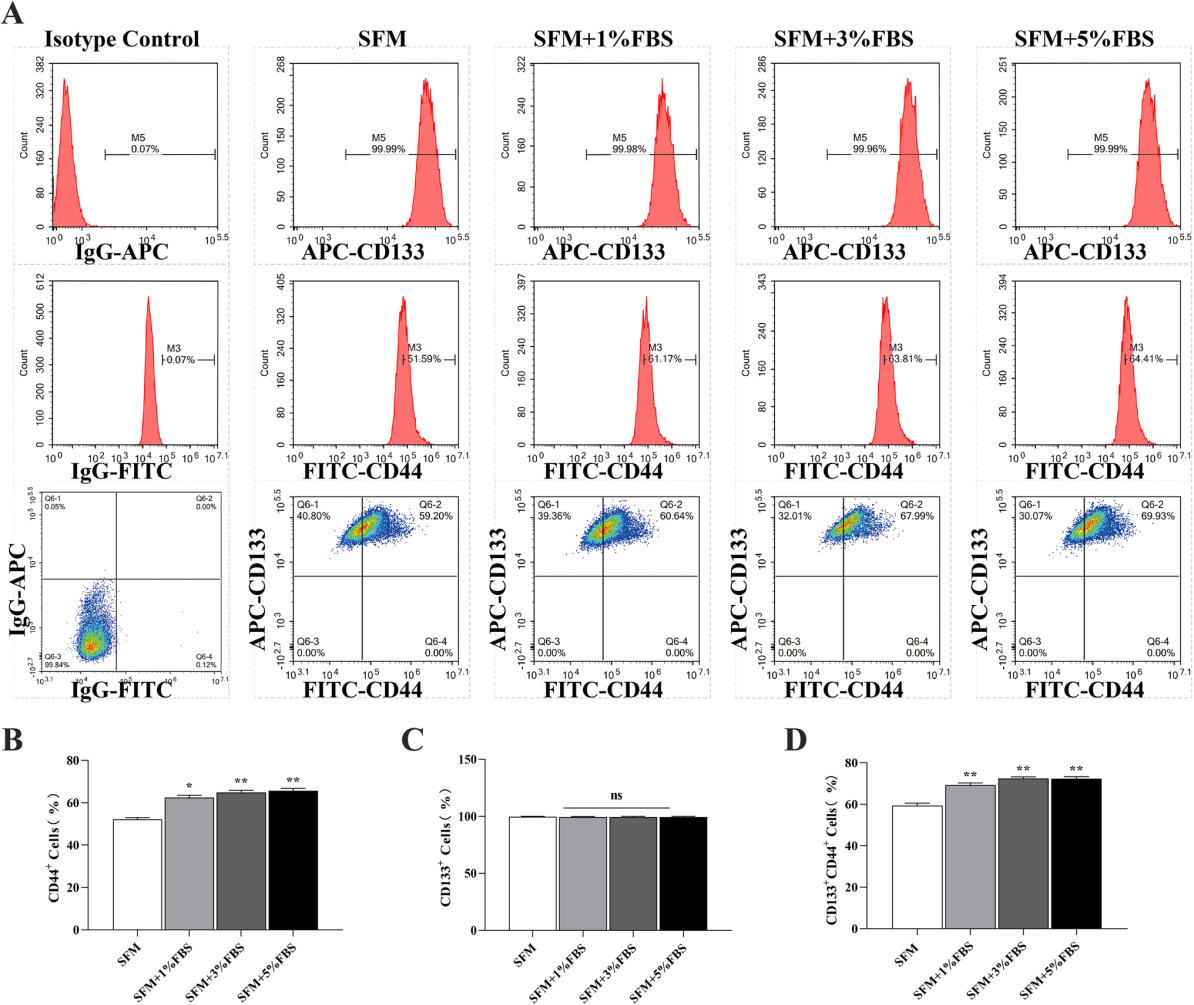
Characterization of stemness phenotype of cancer cell-derived spheres formed in a microserum environment: **A** Fluorescence-activated cell sorting analysis of tumor spheres for CD133+, CD44+, and CD133+/CD44+ cells. **B–D** Proportion of CD44+, CD133+, and CD133+/CD44+ cells. *P < 0.05; **P < 0.01 vs SFM

Our data showed for the first time that expression of the CSC marker CD44 was significantly higher in cells isolated from the serum spheres than in those from serum-free spheres, when the percentage of fetal bovine serum in the culture medium was higher than 1% (Fig. 3A, B). Further, 99% of spheroid cells from serum-free or serum cultures were positive for CD133 expression (Fig. 3C). The CD133^+^CD44^+^ cell fraction was higher in serum sphere cells than in serum-free sphere cells (Fig. 3D).

**Fig. 4:**
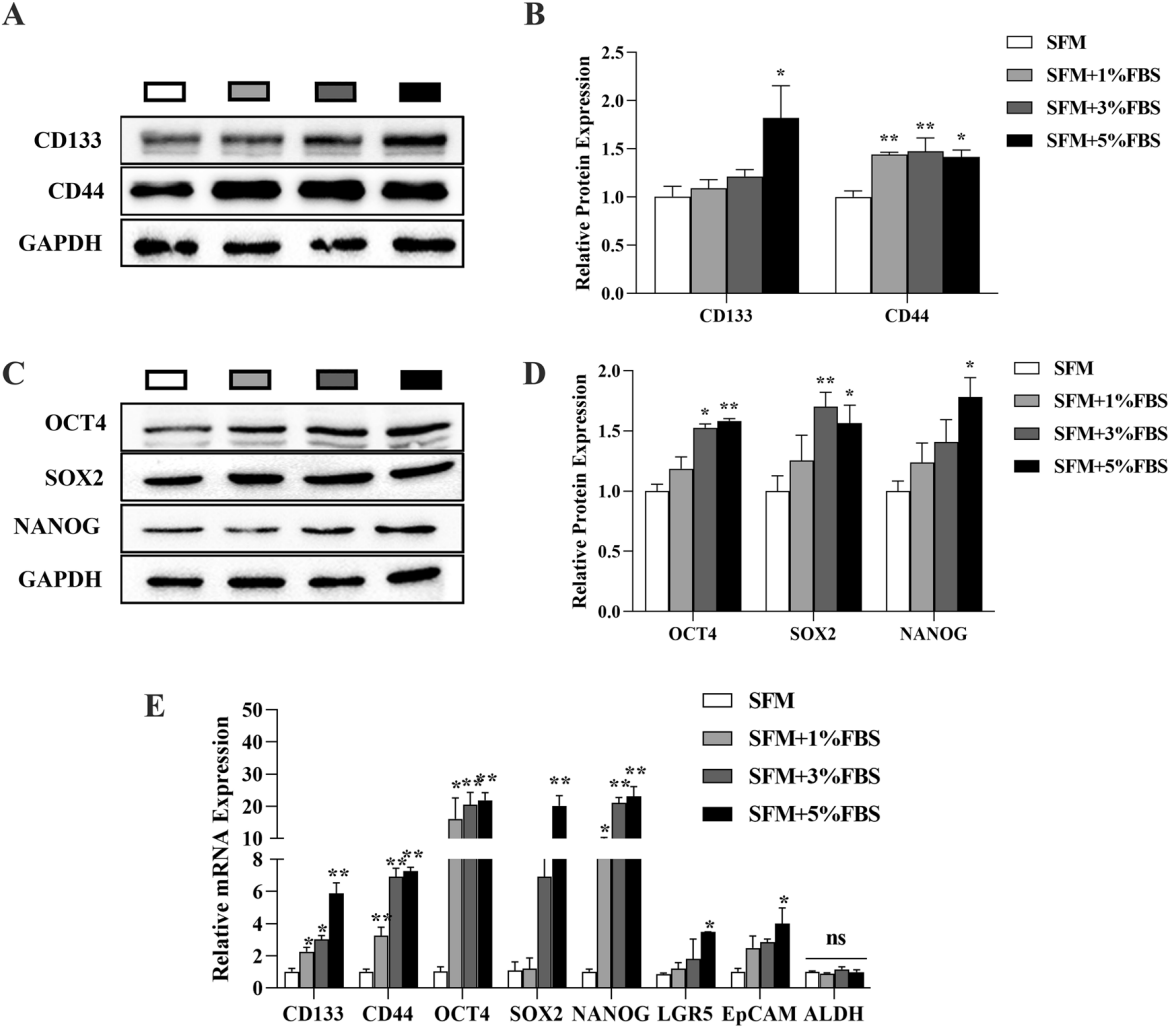
Comparison of the expression of CSC markers and pluripotent stem cell markers in primary spheroids obtained by enriching subpopulations of colon cancer CSCs in a microserum environment: **A**, **B** The expression of the CSC markers CD133 and CD44 was detected by immunoblotting. GAPDH was used as a positive control. **C**, **D** Expression of the pluripotent stem cell markers OCT4, SOX2, and NANOG were detected by immunoblotting. GAPDH was used as a positive control. **E** QPCR analysis of CD133, CD44, OCT4, SOX2, NANOG, EpCAM, LGR5, and ALDH mRNA expression. *P < 0.05; **P < 0.01 vs SFM

To further investigate the effect of the microserum environment on stem-like cell properties, We used WB and qPCR to detect the expression of the key stemness markers SOX2, NANOG, and OCT4, and the CCSC markers CD133, CD44, Lgr5, ALDH, and EpCAM. Comparison of immunoblot analysis showed that the addition of trace amounts of fetal bovine serum elevated the expression of CSC markers (including the commonly expressed CD133, CD44, OCT4, NANOG, and SOX2) during the enrichment of CSCs by colon cancer tumor cells HCT116, and the difference was statistically significant when the percentage of fetal bovine serum in the culture medium was higher than 3% (Fig. 4A–D). qPCR analysis showed that the expression of CD133, CD44, and OCT4 and SOX2, EpCAM, and LGR5 in CSC sphere cells enriched after the addition of micro serum was higher than that in tumor cell spheres enriched in serum-free medium. There was no significant difference in ALDH expression between the groups (Fig. 4E). Key CSC features including the expression of stemness genes and CSC markers were preserved in the microserum environment and increased when the serum concentration was more than 3%.

### Serum culture sphere cells display a higher capacity for proliferation and clone formation

**Fig. 5:**
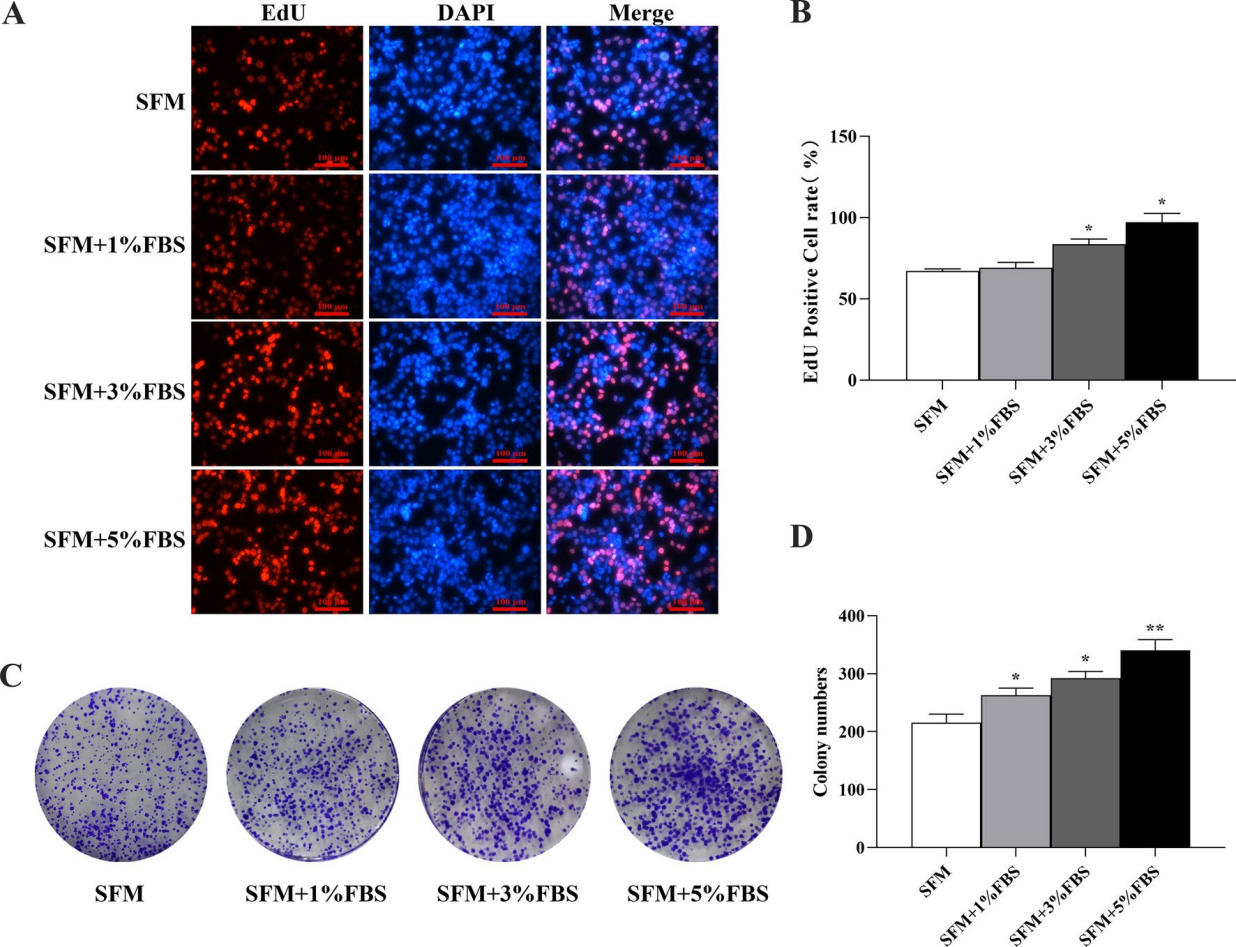
Edu/DAPI fluorescence double staining and plate cloning to compare the proliferation ability of HCT116 spheres cells cultured in a microserum environment and serum-free medium: **A**, **B** Detection of the DNA synthesis ability of serum spheres cells via EdU Staining. The relative EdU-positive cell count in serum spheres and serum-free spheres after adherent culture in complete medium (magnification: 200 ×); **C**, **D** Detection of the proliferative ability of serum sphere cells by colony formation assay. Colonies formed by serum and serum-free spheres after adherent culture in complete medium. *P < 0.05; **P < 0.01 vs SFM

We used EdU staining to determine the DNA synthesis and cell proliferation ability. After adherent culture in complete medium for some time, the relative EdU fluorescence percentage of serum spheres was significantly higher than that of serum-free spheres, and the proliferation rate of serum spheres cells was higher than that of serum-free sphere cells (Fig. 5A, B). Colony formation assays showed that more colonies were formed by microserum environment derived spheres than by spheres enriched in serum-free medium, and the colony forming ability of serum sphere cells was higher than that of serum-free sphere cells. This suggests that CSCs enriched in microserum environment likely have a stronger survival ability in response to environmental changes than do CSCs grown in serum-free conditions (Fig. 5C, D).

### Microserum environment enhanced migration in CSCs isolated from HCT116 spheroids

**Fig. 6:**
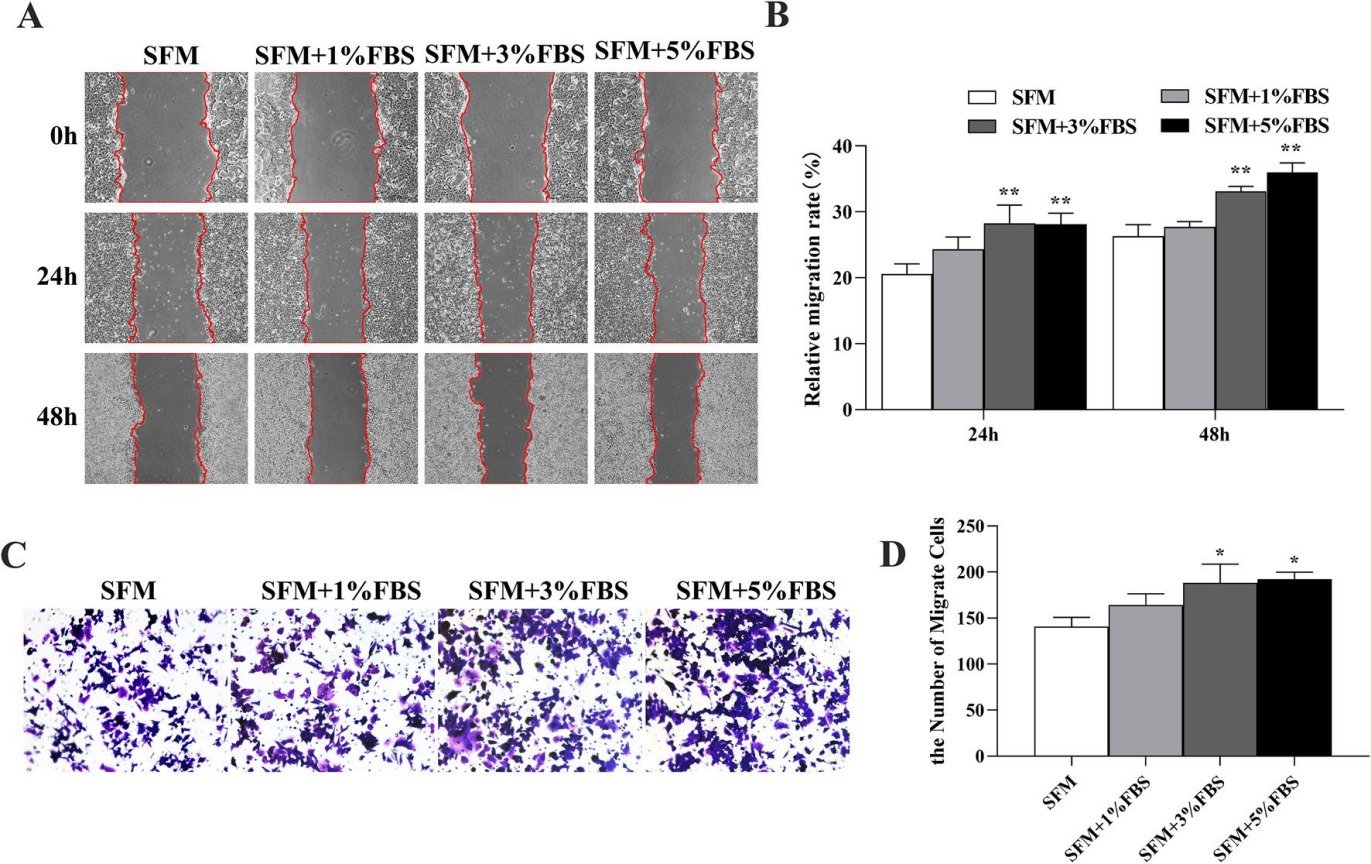
Microserum environment increases the migratory ability of CSCs isolated from HCT116 spheroids: **A**, **B** Wound healing migration assay of HCT116 cells enriched in microserum environment (magnification: 40 ×). **C**, **D** Transwell migratory assay of HCT116 cells enriched in microserum environment (magnification: 100 ×). *P < 0.05; **P < 0.01 vs SFM

To investigate the malignant profile of spheroid cells, we performed transwell invasion assays as well as a wound healing assay to compare the migratory/invasive capacity of serum sphere cells and serum-free sphere cells. Serum sphere-derived cells had significantly higher rates of invasion than did serum-free sphere-derived cells. The wound healing assay results suggested that the migration ability of the serum-free sphere cells was significantly better than that of the serum-free sphere cells at 24 h and 48 h. These data suggest that the serum sphere cells are more malignant than serum-free sphere cells (Fig. 6).

### Microserum environment promotes the migration of cancer cells by driving tumor spheroids toward EMT

**Fig. 7:**
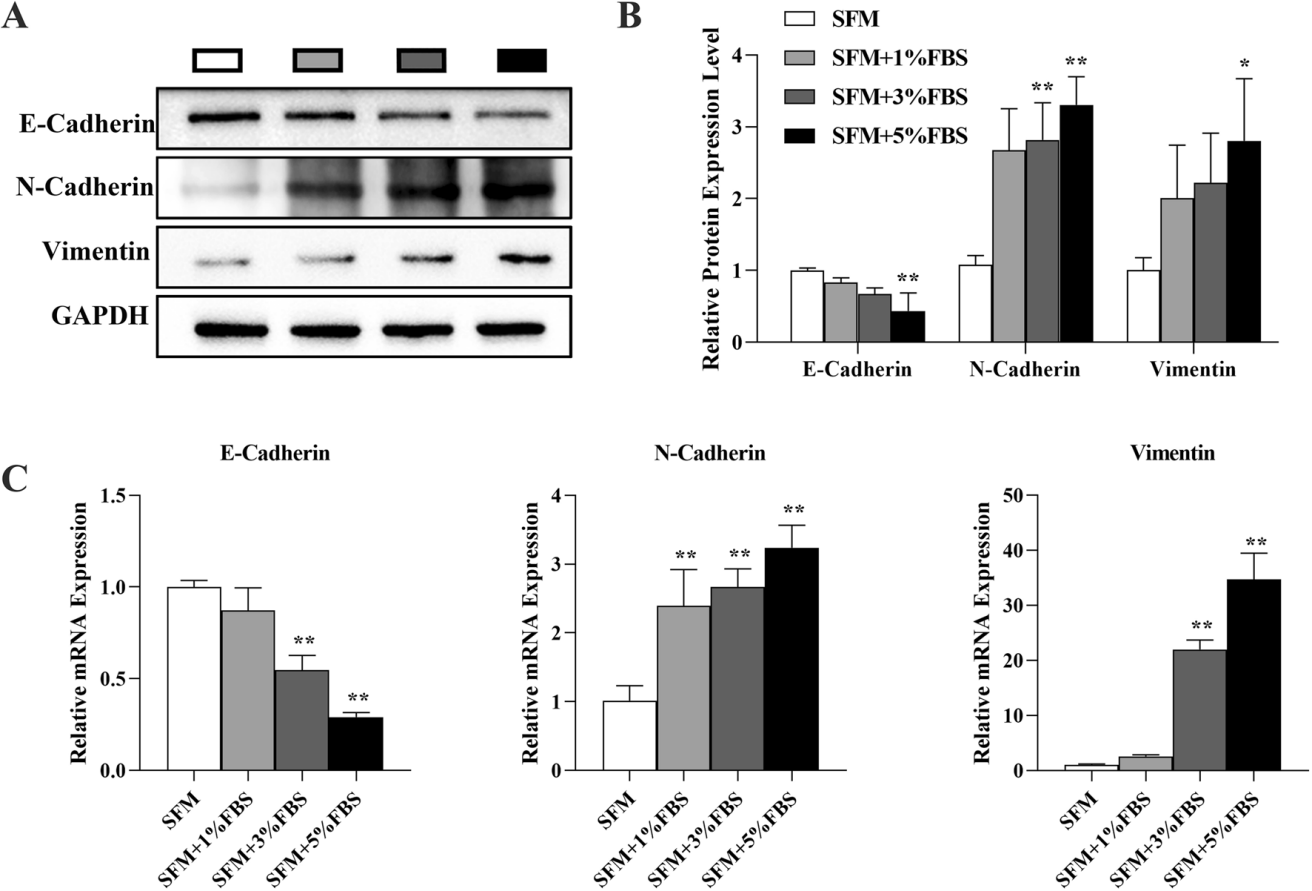
Microserum environment promotes the migration of cancer cells by driving tumor spheroids toward EMT: **A** Western blot analysis of E-Cadherin, N-Cadherin, and Vimentin expression. **B** Quantification of E-Cadherin, N-Cadherin, and Vimentin expression from western blots. **C** QPCR analysis of E-Cadherin, N-Cadherin, and Vimentin mRNA expression. *P < 0.05; **P < 0.01 vs SFM

Vimentin and N-Cadherin expression was upregulated and E-Cadherin expression was downregulated in tumor cell spheres enriched from HCT116 cells after an increase in serum concentration (Fig. 7). These results indicated that a microserum environment promotes the migration of cancer cells by driving tumor spheroids toward EMT. These results validated that adding a small amount of serum could promote the migration and invasion of CSCs in the process of enriching tumor cell spheres.

### Serum spheroid cells contain cytokines and induce spheroid formation and stem-like cell properties and migration in CCSCs

**Fig. 8:**
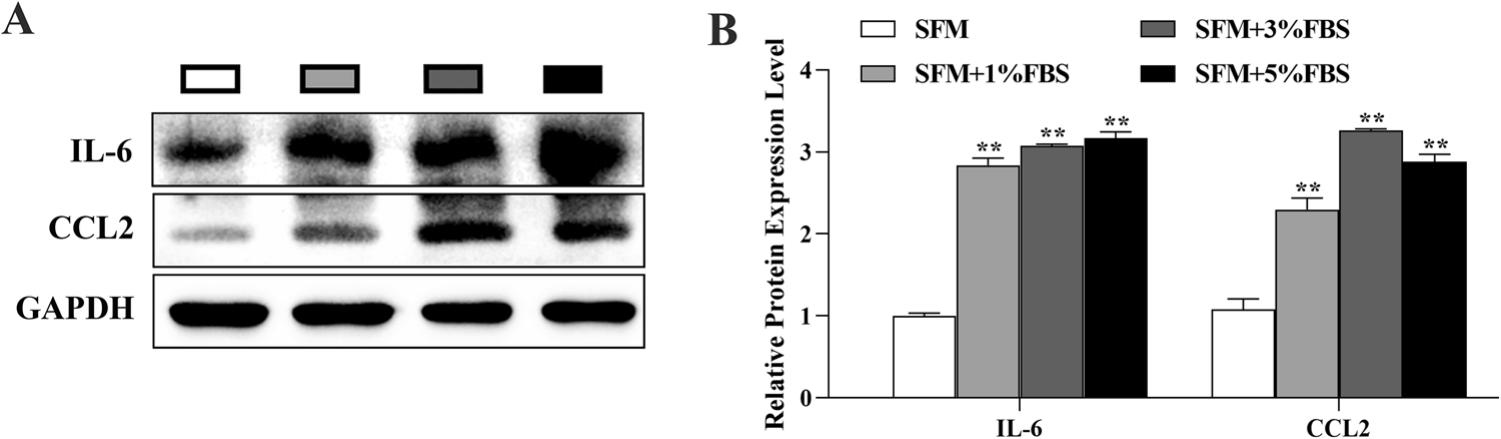
Serum spheroid cells contain cytokines and promote spheroid-formation, stem-like cell properties, and migration in CCSCs: **A** Western blot analysis of IL-6 and CCL2 protein expression. **B** Quantification of IL-6 and CCL2 expression from western blots. *P < 0.05; **P < 0.01 vs SFM

To elucidate the mechanisms by which a microserum environment promotes spheroid formation, stem-like cell properties, and migration of CCSCs, the expression of inflammatory cytokines and chemokines were analyzed in protein lysates from tumor spheres grown in a microserum environment. Serum spheroids expressed significantly higher levels of IL-6 and CCL2 than did serum-free spheroids (Fig. 8A, B).

### Serum spheroid cells enriched in a microserum environment have high tumorigenic and metastatic potential in vivo

We cultured the same number of HCT116 cells as sphere cells under corresponding conditions (adherent culture in intact medium, suspension culture in serum-free medium, suspension culture in microserum environment). When the number of HCT116 cells in adherent culture reached 1 × 10^6^, the number of cells in the sphere cultured in serum-free medium was 2 × 10^5^. The number of cells in the sphere suspended in serum-free medium containing 1% fetal bovine serum was 2.25 × 10^5^, the number of cells in the sphere suspended in serum-free medium containing 3% fetal bovine serum was 2.5 × 10^5^, and the number of cells in the sphere suspended in serum-free medium containing 5% fetal bovine serum was 2.5 × 10^5^. Thus, different numbers of tumor cells were obtained under different culture conditions in cultures with the same initial number of cells.

**Fig. 9:**
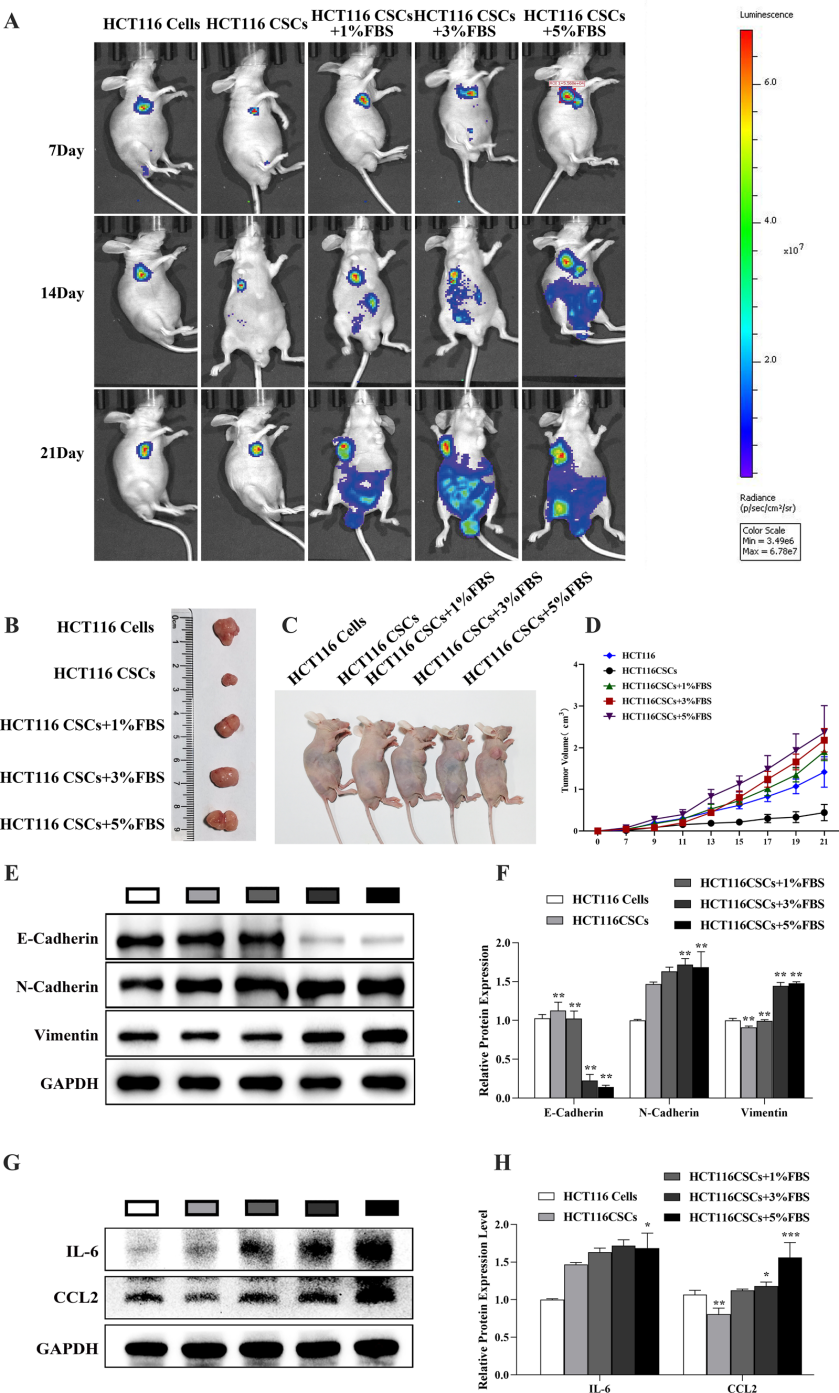
Comparison of tumor formation capacity of HCT116 cells derived from serum spheres, serum-free spheres, and adherent culture: **A** Tumor growth and metastasis were monitored by bioluminescence measurements performed with a high-sensitivity CCD imager. **B–D** Photographs of representative severe combined immunodeficiency mice bearing the subcutaneous xenograft tumors formed by the inoculated HCT116 cells derived from serum spheres, serum-free spheres, or adherent culture. **E** Western blot analysis of E-Cadherin, N-Cadherin, and Vimentin protein expression in tumor tissues. **F** Quantification of E-Cadherin, N-Cadherin, and Vimentin expression from western blots. **G** Western blot analysis of IL-6 and CCL2 protein expression in tumor tissues. **H** Quantification of IL-6 and CCL2 expression from western blots. *P < 0.05; **P < 0.01 vs control

High tumor formation capacity is a major characteristic of CSCs. We therefore performed an in vivo tumorigenicity assay to evaluate the tumor formation capacity of HCT116 cells cultured under different conditions (Fig. 9A). The results showed that at quantities ≤ 3 × 10^5^, after subcutaneous injection into the right armpit of nude mice, except for HCT116 cells in serum-free spheres, the tumorigenicity of sphere cells enriched in trace serum was not less than 1 × 10^6^ adherent cells. Tumor cell spheres enriched in trace serum were rich in CCSCs and showed higher tumor initiation ability than did those without serum or adherent cultured cells. Bioluminescence intensity evaluated as total flux (photons per second), started to develop differently from day 15 of the experiment for serum sphere cells, serum-free sphere cells, and adherent cells xenografts; bioluminescence showed that the serum globular cell group had severe abdominal metastasis, and the overall condition of the mice in the serum sphere cells group were worse than that of mice in the serum-free sphere cells and adherent cells groups. Some mice of the serum sphere cells group showed ascites and severe emaciation.

At the termination of the experiment, the tumor sizes were determined after the mice were sacrificed. For tumor volume, all mean values from tumors in the serum spheres group were considerably higher than those from the serum-free spheres group. However, the differences lacked statistical significance because of high variance (Fig. 9B–D). Western blot analysis demonstrated that the content of E-cadherin in serum globular tumor tissue decreased, and the expression of N-cadherin and vimentin increased in a murine xenograft model of CRC (Fig. 9E, F). Levels of inflammatory cytokines and chemokines, including IL-6 and CCL2, in tumor cells increased in serum globular tumor tissues, which showed an increase in tumor cells. These results were consistent with the results of in vitro studies (Fig. 9G, H).

## Discussion

In this study, we tested a novel system to culture CCSCs. We initially used several concentrations of FBS (10%, 5%, 3%, and 1%), and three colon cancer cell lines (HCT116, LOVO, and SW620) to determine which systems would yield parental colon cancer tumor cells to form spheres. SW620 and LOVO cells could not effectively form spheres when the serum concentration in the culture system was higher than 1%. Further, FBS concentrations < 5% were optimal for sphere formation in HCT116, and other concentrations did not effectively promote the sphere formation. In the ≤ 5% FBS microserum environment, spheres were produced faster and were larger, spherical or rounded, and had smooth contours. CSCs extracted from the microserum environment had enhanced renewal ability; the cells showed a geometric increase and maintained their essential stem cell‐like properties. This new culture method could circumvent some weaknesses of cancer stem-like cell cultures in systems with bFGF, EGF, and B27, which are ineffective, time-consuming, and costly [24].

We focused on challenges surrounding the preservation of key CSC features. CD133 and CD44 are the most frequently reported CSC markers for colorectal cancer [25–27]. To determine whether the spheres were enriched with CCSCs, we analyzed the expression of CD133 and CD44. The proportion of CD44+ cells in serum-free spheres was smaller than that in serum sphere cells. There was no significant difference in the expression of CD133 between the serum-free sphere and serum sphere cells. The percentage of CD133^+^/CD44^+^ cells increased significantly (from 59.2% to 69.53%) in serum sphere cultures. This indicates that high-quality CSCs could be induced, extracted under a microserum environment. Nanog, SOX2, and OCT4 are important transcription factors that modulate the self-renewal and proliferation of embryonic stem cells [28]. Nanog, SOX2 and OCT4 are overexpressed in various types of CSCs and are the main regulators of pluripotency that maintain the undifferentiated state and self‐renewal capacity of CSCs [29–33]. Lgr5, ALDH1, and EpCAM also play a crucial role in the maintenance of CSCs and are potential therapeutic targets for colon cancer [34–36]. Our results showed the upregulation of typical CSC markers including CD133, CD44, Lgr5, ALDH1 and EpCAM, SOX2, NANOG, and OCT4 with the increase of serum concentration in the medium in colon cancer tumor cell line HCT116 cell spheres compared with cell spheres enriched in serum-free medium alone. Different tumor markers may reflect different functions of CCSCs and provide more targets for study and therapy [37]. Moreover, these markers are closely related to tumorigenesis, cancer metastasis and recurrence [38].

In contrast to monolayers, cells cultured into spheroids exhibit a three-dimensional structure that includes the necrotic core, the peripheral proliferating cell layer, and the quiescent cells sandwiched in between, which may alter intercellular interactions. Compared with the outer layer cells, the cells inside the sphere are less stimulated and affected by the outside environment [39]. However, the cells inside the sphere receive less nutrition, the addition of serum in small amounts can replenish the nutrients required by the inner cells. Therefore, the serum-containing medium may be better suited for the conversion of inner layer cells into quiescent or dormant cells, which are more stem-like [40].

The induction of many cancers, including pancreatic, colon, liver lung and ovarian cancers, is closely related to the EMT process [41]. It has been shown that being in the EMT state is more likely to enrich cells that exhibit stem cell properties [42], These cells show similar characteristics to stem cells isolated from normal or tumor cell populations [43]. When some factors induce the expression of EMT markers, they also upregulate CSC markers and promote the acquisition of stemness, tumorigenesis and metastasis [44, 45]. SOX2 was reported to activate autophagy by transcriptionally promoting Beclin1 expression, thereby upregulating EMT-related genes and promoting sphere enrichment and cancer progression [46]. The serum globules of HCT116 cells and xenograft tumors formed by subcutaneous inoculation of serum globules exhibited higher EMT expression than did the serum-free spheres. We verified in vivo and in vitro that HCT116 spheres expressed higher migratory/invasive capacity and tumor formation capacity, and serum spheroids were more malignant than serum free spheroids. Therefore, we speculate that the high metastatic ability of serum spheres is due to EMT of CSCs induced by the addition of low concentrations of serum during enrichment. This process promotes CCSCs to exhibit higher Sphere formation and proliferation abilities. Our results indicate that the microserum environment induced the formation of CCSC spheroids, promoted stem cell characteristics, and increased migration, which was caused by a small amount of serum changing the tumor microenvironment and promoting the expression of related cytokines.

Chemokines are closely related to the proliferation, invasion, and migration processes of tumor cells [47, 48]. Chemokine (C–C pattern) ligand 2 (CCL2) regulates progression to cells in the tumor microenvironment as well as cancer [49, 50]. In animal models and patients, IL-6 is a key inflammatory mediator in the development and progression of colorectal cancer [51, 52]. After treatment with exogenous IL-6, Wang et al. observed enhanced drug resistance in spheroid cells, along with increased expression of their tumor stem cell-associated genes [53]. Our data support the notion that the tumor microenvironment, in combination with serum, stimulates CSCs to secrete cytokines, such as IL-6 and CCL2, that act paracrine to cancer cells to enhance their malignant properties, including sphere formation, stem cell characteristics, and migration ability [54].

## Conclusion

Our study provides evidence that the HCT116 colon cancer cell line can retain stem cell properties and the multi-potential ability can be restored under specific conditions. Adding a certain concentration of serum to serum-free medium is beneficial for the enrichment of CSCs. The addition of a small amount of serum can enhance the expression of inflammatory factors and chemokines in CSCs, and trigger EMT, which gives HCT116 cells stronger spheroidizing ability and stem cell characteristics. This system could provide a rapid and economical method for generating CCSCs and provide new insights and new models for CCSC research.

## Data Availability

The datasets used and/or analyzed during the current study are available from the corresponding author on reasonable request. All data generated or analyzed during this study are included in this published article.

## Abbreviations

CSCs: Cancer stem cells
CCSCs: Colorectal cancer stem cells
WB: Western blot
QPCR: Quantitative polymerase chain reaction
EdU: 5-Ethynyl-2′-deoxyuridine
EMT: Epithelial–mesenchymal transition
FACS: Fluorescence-activated cell sorting
MACS: Magnetic-activated cell sorting
SP: Side population
SF: Serum-free
DMEM: Dulbecco’s Modified Eagle’s Medium
FBS: Fetal bovine serum
SFE: Sphere‐forming efficiency
PBS: Phosphate-buffered saline
ECL: Enhanced chemiluminescence
SD: Standard deviation
CCK-8: Cell Counting Kit-8
CRC: Colorectal cancer
TBST: Tris-buffered saline Tween
3D: Three-dimensional
CCL2: Chemokine C–C motif ligand 2
MCP-1: Monocyte chemotactic protein-1
